# Epitranscriptomic Analysis of the Ventral Hippocampus in a Mouse Model of Post-Traumatic Stress Disorder Following Deep Brain Stimulation Treatment of the Basolateral Amygdala

**DOI:** 10.3390/brainsci15050473

**Published:** 2025-04-29

**Authors:** Mingxi Ma, Hao Fan, Hui Zhang, Yao Yin, Yizheng Wang, Yan Gao

**Affiliations:** 1 Center of Cognition and Brain Science, Beijing Institute of Basic Medical Sciences, Beijing 100850, China; mamingxi@fmmu.edu.cn (M.M.); francis_fanhao@163.com (H.F.); yinyao1986@126.com (Y.Y.); yzwang@ion.ac.cn (Y.W.); 2 Department of Neurology, The First Affiliated Hospital of Dalian Medical University, Dalian 116011, China; m13942091644_1@163.com

**Keywords:** PTSD, DBS, vHPC, m^6^A, methylation levels

## Abstract

Background: Basolateral amygdala (BLA) deep brain stimulation (DBS) has been shown to alleviate the symptoms of post-traumatic stress disorder (PTSD), but the specific mechanisms remain incompletely understood. The hippocampus, a brain region closely connected to the amygdala, plays a key role in the pathological processes of PTSD. The N6-methyladenosine (m^6^A) methylation of RNAs in the hippocampus is known to play a significant role in regulating the brain’s response to stress and emotional disorders. Methods: This study aimed to comprehensively analyze the roles of transcriptome-wide m^6^A modifications of the hippocampus in the BLA DBS treatment of a PTSD mouse model using m^6^A sequencing. Results: Significant alterations in functional connectivity between the ventral hippocampus (vHPC) and BLA were observed in foot shock (FS) mice through functional magnetic resonance imaging (fMRI) analysis. Furthermore, we observed that the expression of the key m^6^A methyltransferase enzyme, METTL3, in the FS and BLA DBS groups was higher than that in the control group. At the same time, both FS and BLA DBS induced the widespread m^6^A methylation of RNAs in the vHPC. Gene ontology (GO) enrichment analysis revealed that FS altered methylation in metabolic, developmental, and cytoskeletal pathways, while BLA DBS targeted metabolic, cell cycle, and neuroplasticity-related genes. Additionally, BLA DBS reversed the aberrant methylation of genes associated with multiple functional pathways induced by FS, including those related to cholinergic transmission, sodium and calcium ion homeostasis, and stress hormone responsiveness. We identified a set of RNAs with methylation changes that were reversed by BLA DBS in the FS vs. Ctrl (control) comparison, including those associated with cholinergic transmission, sodium and calcium ion balance, and stress hormone response. Additionally, we detected several specific BLA DBS-related genes through MeRIP-qPCR, indicating that DBS influences crucial genes linked to calcium signaling and synaptic plasticity. Conclusions: We draw two conclusions from these findings: BLA DBS may alleviate PTSD-like symptoms by reversing FS-induced methylation changes and by altering the methylation levels of crucial genes. These findings indicate that epigenetic m^6^A modifications in the vHPC may play an important role in the amelioration of PTSD using BLA DBS.

## 1. Introduction

Post-traumatic stress disorder (PTSD) is a severe mental disorder characterized by a delayed onset and persistent symptoms that occur after an individual experiences, witnesses, or is confronted with a traumatic event [1]. In clinical practice, psychological therapy and pharmacotherapy are commonly used to treat PTSD, but their effectiveness is often limited, especially in treatment-resistant cases [1,2,3].

DBS is a neurosurgical procedure that enables targeted circuit-based neuromodulation and has demonstrated substantial efficacy across a spectrum of neurological and psychiatric disorders [4,5]. DBS has been applied in the treatment of various neuropsychiatric disorders including depression, obsessive compulsive disorder (OCD), bipolar disorder, and PTSD [6,7]. For example, a meta-analysis found that the standardized Y-BOCS scores of over 35% of patients reduced by more than 35% after DBS treatment [8]; another meta-analysis demonstrated that DBS exhibited significant efficacy in treating TRD, with a standardized mean difference (SMD) of 0.75, indicating superior effectiveness compared to the control group [9]. Furthermore, clinical findings indicate that the dysregulation of amygdala activity plays a central role in PTSD, while BLA DBS has shown promising therapeutic potential for treatment-resistant PTSD cases [10,11], offering new hope for patients who do not respond to conventional therapies. However, the exact mechanisms of its therapeutic effects remain to be fully elucidated.

The hippocampus, a critical brain structure involved in memory formation and emotional regulation, has emerged as a key region in PTSD pathophysiology. A meta-analysis of structural MRI studies revealed a consistent bilateral hippocampal volume reduction in adults with PTSD [12], highlighting the structural alterations associated with the disorder. Additional studies have revealed a positive correlation between the reduced left hippocampal volume and PTSD symptom severity [13], with specific subfield atrophy (e.g., CA1) potentially linked to clinical severity [14,15]. However, inconsistencies exist across hippocampal volumetry findings in PTSD research. While some studies report significant whole-hippocampal volume reductions in patients, others fail to demonstrate such differences [16]. These discrepancies may stem from heterogeneous sample characteristics (e.g., trauma type, sex, age, etc.).

Noteworthily, the genes expressed in the dorsal hippocampus (dHPC) are associated with cortical areas involved in information processing, whereas the genes expressed in the ventral hippocampus (vHPC) are linked to regions regulating emotions and stress [17]. The dHPC is primarily involved in the formation and retrieval of contextual memories, whereas the vHPC plays a more prominent role in emotional memory processing and fear responses [18]. This functional dichotomy likely underlies their distinct contributions to PTSD pathophysiology. In particular, the ventral hippocampus—a subregion that is recognized for its role in stress regulation and emotion processing—has garnered increasing attention as a key contributor to the development and progression of PTSD [19]. The interaction between the ventral hippocampus and the basolateral amygdala is critical for the appropriate regulation of fear and emotional memory. In PTSD, the balance between these two structures is often disrupted. Research has indicated that the activation of BLA-vHPC synapses can rapidly and significantly amplify anxiety-related behaviors, whereas their inhibition mitigates such behaviors [20].

Epigenetic mechanisms play a role in the detrimental effects of traumatic stress and the development of PTSD [21]. N6-methyladenosine (m^6^A), the most prevalent internal modification of RNA, has garnered significant attention for its crucial roles in neural plasticity, learning and memory, and stress responses, driven by advancements in sequencing technologies such as MeRIP-seq. Multiple studies have demonstrated close associations between m⁶A modifications and stress responses/psychiatric disorders (e.g., depression). Changes in m^6^A/m dynamics have been observed in depressed patients and stressed mice, highlighting the significance of m^6^A/m modifications in stress-related psychiatric disorders [22]. Tricyclic antidepressants (TCAs) have been found to increase the FTO expression and activate its epigenetic function in the ventral tegmental area (VTA), leading to antidepressant effects. Additionally, the overexpression of FTO in the VTA or m^6^A modifications in the VTA have shown significant antidepressant effects [23]. The inhibition of astrocyte ALKBH5 expression in the medial prefrontal cortex (mPFC) has also produced antidepressant effects [24].

In the vHPC, m^6^A modifications influence neuronal function, learning and memory [25], emotional regulation, and stress responses [26] through the regulation of RNA metabolism and gene expression. In recent years, the role of m^6^A modifications in the pathogenesis of PTSD has garnered increasing attention. Studies have explored the use of N-acetylcysteine (NAC) as a potential treatment for PTSD, and they have found that NAC can reduce the m^6^A methylation levels and increase the ATF4 expression in the hippocampus [27]. This leads to improvements in cognitive function and reduces neuronal apoptosis in mice. These findings suggest that the dynamic regulation of m^6^A in the hippocampus is closely associated with the pathogenesis and progression of PTSD. Furthermore, the role of m^6^A modifications in the vHPC in the therapeutic efficacy of BLA DBS for PTSD warrants further investigation.

Inescapable foot shock (FS), a widely employed mouse model of PTSD [28], was employed in this study. BLA DBS transcriptome-wide m^6^A changes were profiled using immunoprecipitated methylated RNAs with microarrays in the control, FS, and BLA DBS groups to investigate the molecular mechanisms of BLA DBS action in the vHPC for PTSD treatment. By conducting a thorough comparative analysis, we identified key genes and signaling pathways in the vHPC that could be involved in the improvement of PTSD-like behavior through BLA DBS. Our study offers fresh insights into the epigenetic regulation of the vHPC in PTSD treatment with BLA DBS, shedding light on its involvement in the development of PTSD.

## 2. Materials and Methods

All animal experiments and procedures were conducted in accordance with protocols approved by the Institutional Animal Care and Use Committee (IACUC) of the Beijing Institute of Basic Medical Sciences. Mice were group-housed (4–5 animals per cage) under standard conditions with ad libitum access to food and water. The housing facility maintained a 12-h light–dark cycle (lights on at 7:00 a.m. and off at 7:00 p.m.). Six- to seven-week-old male C57BL/6 mice weighing 20–22 g were utilized for all experimental procedures.

### 2.1. Electrode Implantation

To embed electrodes, mice were fixed in a stereo locator, and they were placed on the skin under anesthesia. A 3% hydrogen peroxide solution was used to clean the skull surface and expose the perioperative area and anterior fontanelle spot. The coordinates for the median basolateral amygdala (BLA) on both sides were determined using a brain anatomical map. Holes were drilled into the skull at the coordinates using a cranial drill. The electrode was slowly inserted vertically alongside the BLA (−1.34 mm AP, ±3.3 mm ML, −4.8 mm DV from the brain surface). Following anesthesia, the mice were placed on a heating pad to recover. Once fully awake, they were relocated to their home cages for 7 days to recover.

### 2.2. Inescapable Foot Shock

Three days prior to the commencement of the experiment, mice were habituated to the experimental environment by being placed within it for 10 min daily. Mice were placed in the experimental room for acclimation at least 30 min prior to the start of the procedure. Electric foot shocks were delivered using a fear-conditioning chamber (35 cm × 20 cm × 20 cm) (Panlab, Spain) at an intensity of 0.8 mA [29,30]. The shock procedure involved allowing the mice to move freely within the chamber for 5 min, followed by a total of 16 intermittent and inescapable foot shocks, with each shock lasting for 10 s and separated by 10 s intervals [31,32]. On the second day, the stimulation was repeated to consolidate fear memories and increase the likelihood of PTSD-like behaviors emerging in the mice [33]. Mice in the control group were placed in the chamber for 10 min without any stimulation. To prevent the influence of feces and odors, the chamber was wiped with a 75% ethanol solution before each mouse began the experiment.

### 2.3. Deep Brain Stimulation

We performed DBS using the Master 8 stimulator (A.M.P.I., Jerusalem, Israel) and adjusted the stimulation frequency and intensity via the stimulator isolator settings. DBS was commenced 24 h following the final FS, administering 100 μA, 100 μs, and 130 Hz DBS to the animals for 1 h daily, spanning a week. DBS was initiated 24 h following the final FS session, with parameters set at 100 μA current, 100 μs pulse duration, and 130 Hz frequency, administered for 1 h per day over a 7-day period. The selection of these parameters was based on their demonstrated efficacy in prior studies [34], their ability to generate charge densities comparable to those used in clinical DBS treatments [10,35], and the foundation laid by our previous research [29].

### 2.4. m^6^A Epitranscriptomic Microarray Analysis

Upon the completion of the final behavioral assessment, the mice were subjected to saline perfusion, and their brains were meticulously dissected. Utilizing a cryostat set at −20 °C, the brains were sectioned at the targeted region. The precise extraction of the BLA tissue was achieved with a 1 mm round tissue punch, and the specimens were promptly preserved at −80 °C. Each analytical sample constituted a composite from ten individual mice. The quantification of total RNA was executed via the NanoDrop ND-1000 spectrophotometer (Thermo Fisher Scientific, Waltham, MA, USA). The preparation of samples and the process of microarray hybridization adhered to the standardized protocols established by Arraystar. In summary, total RNAs underwent immunoprecipitation with an m^6^A antibody. The m^6^A-methylated RNAs were subsequently eluted from the magnetic beads, constituting the “IP” fraction, whereas the unmodified RNAs were retrieved from the supernatant, forming the “Sup” fraction. These “IP” and “Sup” RNA fractions were distinctly labeled with Cy5 and Cy3 fluorophores, respectively, employing the Arraystar Super RNA Labeling Kit to synthesize cRNAs. The amalgamated labeled cRNAs were then hybridized onto the Arraystar Mouse mRNA & lncRNA Epitranscriptomic Microarray (8 × 60K, Arraystar, Rockville, MD, USA). Post-hybridization, the microarray slides were cleansed and scanned across dual-color channels with the Agilent Scanner G2505C (Agilent Technologies, Santa Clara, CA, USA). The resultant array images were scrutinized using the Agilent Feature Extraction software (version 11.0.1.1). The raw fluorescence intensities corresponding to the IP and Sup fractions were normalized against the average intensities of log2-transformed Spike-in RNA controls. After Spike-in normalization, probe signals that exhibited present (P), marginal (M), or quality control (QC) flags in at least one of the triplicate samples were selected as “All Targets Value” within the Excel datasheet, paving the way for the subsequent analyses of “m^6^A methylation level”, “m^6^A quantity”, and “expression level”.

### 2.5. Western Blot

Mice were anesthetized with 0.7% sodium pentobarbital (70 mg/kg), and the brain was removed quickly. Brain tissue containing the vHPC was excised under a stereomicroscope from 3 mice per group (pooled samples), placed in EP tubes, and mixed with 50 μL of strong RIPA lysis buffer. The samples were placed on ice for 30 min to fully lyse the cells. Then, 50 μL of 2× SDS was added, mixed well, and heated at 100 °C for 10 min using a metal bath heating block. The samples were then centrifuged at 12,000× *g* and 4 °C for 30 min. The supernatants were collected for subsequent experiments.

An SDS-8% PAGE gel was prepared, and after loading the protein samples, electrophoresis was performed to separate the proteins. Subsequently, the proteins were transferred onto a polyvinylidene difluoride (PVDF) membrane using the wet transfer method (220 mA, 3 h). The PVDF membrane was blocked at room temperature for 2 h with the blocking buffer (5% skim milk in PBS). After blocking, the target protein bands were precisely cut from the PVDF membrane based on the molecular weight markers and incubated with corresponding rabbit-anti- Mettl3 antibody from Abcam (226017, 1:1000, Shanghai, China) at 4 °C overnight. Following primary antibody incubation, the membranes were washed three times with PBST (15 min per wash). The membranes were subsequently incubated with HRP-conjugated secondary antibodies (C31460100, 1:2000) at room temperature for 2 h and then subjected to another three PBST washes (15 min per wash). Finally, protein bands were visualized using Western HRP Substrate and imaged with a chemiluminescence imaging system. The grayscale values of ECL-developed bands were analyzed and statistically processed using the software ImageJ software (version 1.53k).

## 3. Results

### 3.1. vHPC Is Potentially Intricately Associated with PTSD Mice Model

**Figure 1:**
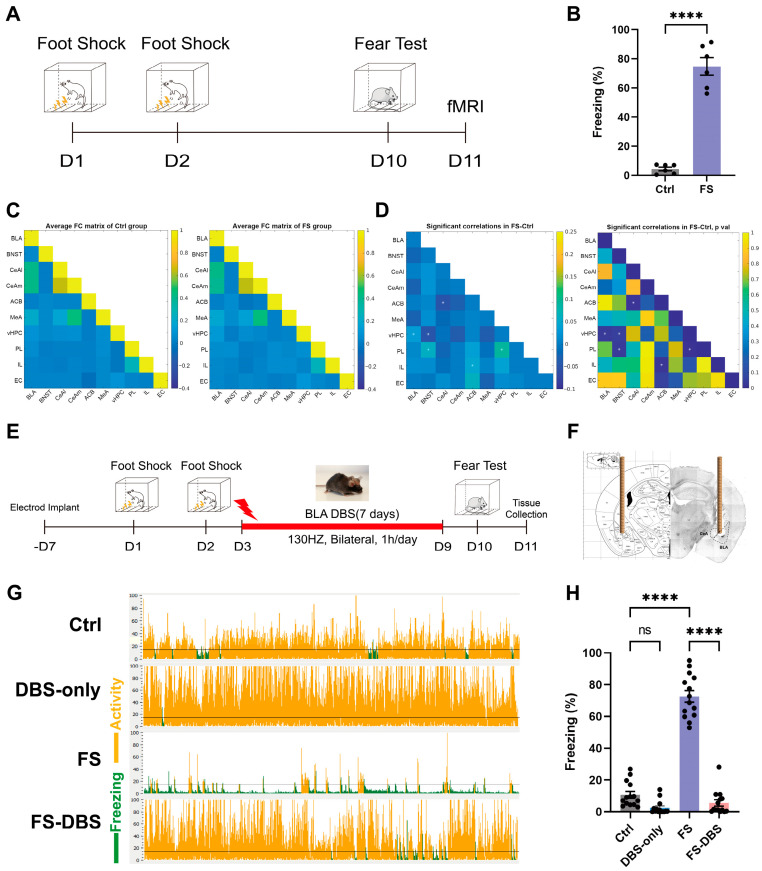
BLA-DBS can effectively improve PTSD-like behaviors in mice. (**A**) The protocol used for inescapable foot shock and fear test. (**B**) Statistics for the fear test (unpaired *t* test, *n* = 6, **** *p* < 0.0001). (**C**) Correlation matrices derived from global fMRI ALFF signal analysis (pseudocolor map of *t* statistics after thresholding at a false discovery rate, *n* = 12, *p* ≤ 0.05) across brain regions in the Ctrl group (left) and FS group (right); warm colors represent higher correlations. (**D**) Difference in functional connectivity between the FS and Ctrl groups (left) with corresponding *p* values (right). * Significant difference (white indicates an increase; unpaired *t* test, *n* = 12, *p* ≤ 0.05). (**E**) The protocol used for BLA-BDS. (**F**) The target site for DBS electrode implantation in the BLA. (**G**) Representative animal tracks of four groups. (**H**) Statistics of the fear memory retrieval test of four groups (one-way ANOVA, *n* = 14, **** *p* < 0.0001).

In clinical practice, BLA DBS has emerged as an effective intervention for patients with treatment-resistant PTSD [1,36]. First, functional magnetic resonance imaging (fMRI) was used to identify brain regions with altered activity associated with the BLA in FS and control mice (Figure 1A). In the contextual fear conditioning test on day 10, the freezing percentage in the FS group was approximately 75%, significantly higher than that in the control group (*p* < 0.0001) (Figure 1B), indicating a robust fear response induced by the FS paradigm. Subsequently, fMRI was used to identify brain regions exhibiting altered activity in the FS model [37,38]. Compared to the control group, mice in the FS group exhibited significantly enhanced functional connectivity between the BLA and vHPC (Figure 1C,D). These findings suggest that the vHPC may serve as an important downstream target of BLA DBS treatment. Furthermore, we observed significantly enhanced functional connectivity between the vHPC and both the bed nucleus of the stria terminalis (BNST) and the prelimbic cortex (PL), between the accumbens (ACB) and both the central amygdala (CeA) and the infralimbic cortex (IL), and between the PL and the BNST. Findings from the PTSD mouse model indicated notable changes in the functional connectivity between the limbic system and the prefrontal cortex, consistent with clinical findings [39,40].

The electrode was inserted vertically alongside the bilateral BLA (Figure 1F). Following the establishment of the FS model, we administered BLA DBS and performed a contextual fear conditioning test on the 10th day of the experiment (Figure 1E). The FS-DBS group showed a significant reduction in freezing behavior compared to the FS group (*p* < 0.0001), with levels returning to those of the control group while the DBS-only group exhibited no difference from the control group (Figure 1G,H). These results demonstrate that a 7-day course of continuous BLA DBS significantly alleviated PTSD-like fear responses in FS-induced mice. At 24 h following the fear conditioning test, mice in the Ctrl, FS, and FS-DBS groups underwent transcardial perfusion with saline and then brain extraction. The vHPC tissue samples were collected using a 1 mm diameter tissue punch in a cryostat and stored at −80 °C for subsequent analysis.

### 3.2. FS and DBS Induced Alterations in m^6^A Methylation Levels in vHPC

**Figure 2:**
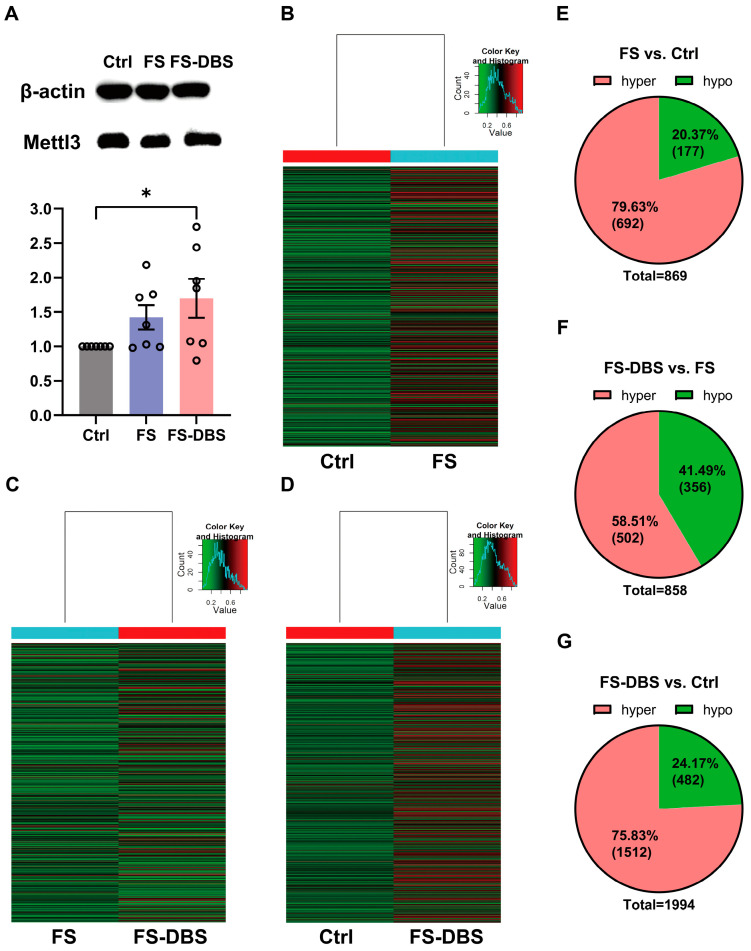
Alteration in the vHPC m^6^A-mRNA methylation levels. (**A**) Western Blot analysis of Mettl3 protein levels in the vHPC of the Ctrl, FS, and FS-DBS groups (above), Western Blot grayscale value statistical graph (below) (one-way ANOVA, *n* = 7, * *p* < 0.05). (**B**–**D**) Heatmap showing genes for which m^6^A-mRNA modification changed by at least 1.5-fold in the FS group compared with the Ctrl group (**B**), the DBS group compared with the Ctrl group, (**C**) and the DBS group compared with the FS group (**D**). (**E**–**G**) Pie chart illustrating the genes with hypermethylated and hypomethylated m^6^A-mRNA methylation levels across (**B**–**D**).

We first measured the levels of Mettl3 (m^6^A writer) using Western blotting and found that the levels of Mettl3 were significantly higher in the FS mice compared to the Ctrl group (42.4% increase), and these levels showed an increasing trend in the FS-DBS group compared to the FS group (19.3% increase) (Figure 2A). Moreover, we examined changes in the m^6^A methylation of RNAs in the vHPC using m^6^A epitranscriptomic microarray analysis, identifying significant alterations with a log2-scaled absolute fold change of ≥1.5. Specifically, compared to the Ctrl group, the FS group showed approximately 80% hypermethylation and 20% hypomethylation (Figure 2B,E). The DBS group showed 60% hypermethylation, while the FS group showed approximately 40% hypermethylation (Figure 2C,F). In the DBS group, relative to the Ctrl group, approximately 75% of methylation was hypermethylated, while 25% was hypomethylated (Figure 2D,G).

The gene expression level exhibited a predominant upregulation pattern, with approximately 60% of genes upregulated and 40% downregulated in the FS group compared to the Ctrl group (Figure S1A,D). In the DBS group, around two-thirds of genes were upregulated and one-third were downregulated, relative to the control group (Figure S1B,E). Additionally, the DBS group had 6537 upregulated genes when compared to the FS group, which was about four times the number of downregulated genes (Figure S1C,F).

### 3.3. Gene Ontology Analysis of m^6^A Modifications in vHPC

**Figure 3:**
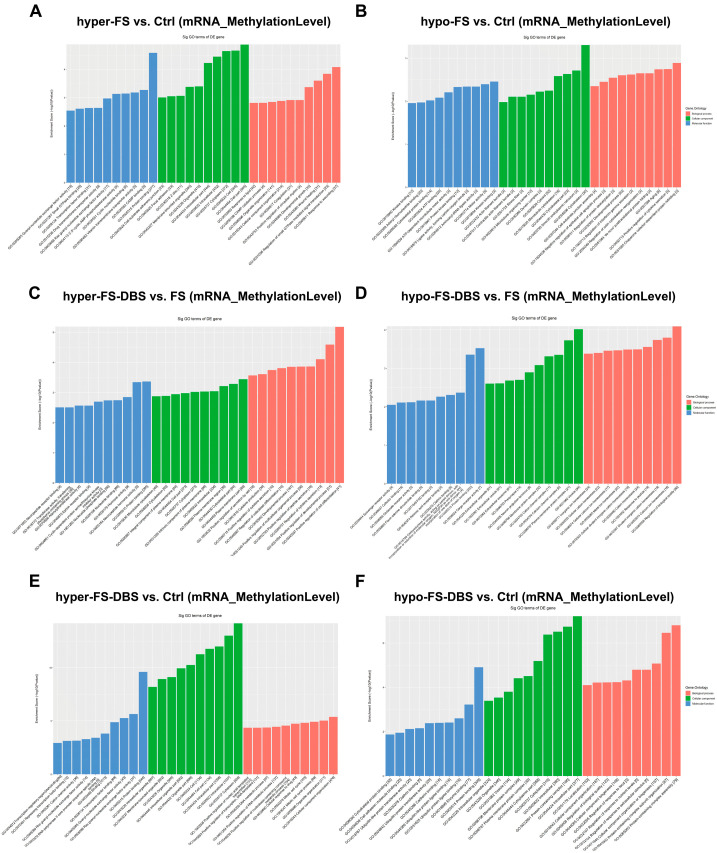
The GO analysis of hypermethylated and hypomethylated m^6^A-mRNAs among the three groups. (**A**,**B**) The GO analysis of hypermethylated (**A**) and hypomethylated (**B**) m^6^A-mRNAs in the FS group compared to the Ctrl group. (**C**,**D**) The GO analysis of hypermethylated (**C**) and hypomethylated (**D**) m^6^A-mRNAs in the FS-DBS group compared to the FS group. (**E**,**F**) The GO analysis of hypermethylated (**E**) and hypomethylated (**F**) m^6^A-mRNAs in the FS-DBS group compared to the Ctrl group.

Additionally, we conducted Gene Ontology (GO) enrichment analysis on different m^6^A-methylated mRNAs across three comparisons (FS vs. Ctrl, FS-DBS vs. FS, and FS-DBS vs. Ctrl), focusing on biological processes, cellular components, and molecular functions (Figure 3 and Table S1). In the FS vs. Ctrl group, the hypermethylated genes were primarily enriched in wound response, developmental growth, and organelle organization (biological processes); intracellular structures and cell junctions (cellular components); and protein binding, signal transduction, and transport activity (molecular functions) (Figure 3A). The hypomethylated genes were mainly associated with cell junction assembly, protein regulation, and developmental processes (biological processes); cytoskeleton and organelle structures (cellular components); and kinase binding, ATP binding, and cytoskeletal protein binding (molecular functions) (Figure 3B).

In the FS-DBS vs. FS group, GO enrichment analysis showed that the hypermethylated genes were primarily associated with cellular secretion, cytokine production, and developmental regulation (biological processes); cytoskeleton, plasma membrane, and cytoplasm (cellular components); and kinase activity, nucleotide binding, and protein binding (molecular functions). The hypomethylated genes were mainly linked to ion homeostasis and the regulation of biological quality (biological processes); extracellular vesicles, membrane protein complexes, and neuron projections (cellular components); and receptor activity, cofactor binding, and protein binding (molecular functions) (Figure 3C,D).

GO enrichment analysis of the FS-DBS vs. Ctrl group revealed that the hypermethylated genes were primarily associated with transcriptional regulation, cell cycle, and organelle organization (biological processes), as well as transcriptional regulation, protein binding, and ion channel activity (molecular functions). The hypomethylated genes were mainly linked to cellular component organization, protein complex assembly, and localization (biological processes), and cytoskeletal protein binding, ubiquitination, and protein binding (molecular functions) (Figure 3E,F). The results suggest that BLA DBS induces a dual regulatory mechanism in m^6^A modifications. The hypermethylated genes promote repair and anti-inflammatory responses, while the hypomethylated genes support ion homeostasis and synaptic function.

### 3.4. BLA DBS Attenuated m^6^A Hypermethylation Caused by FS

**Figure 4:**
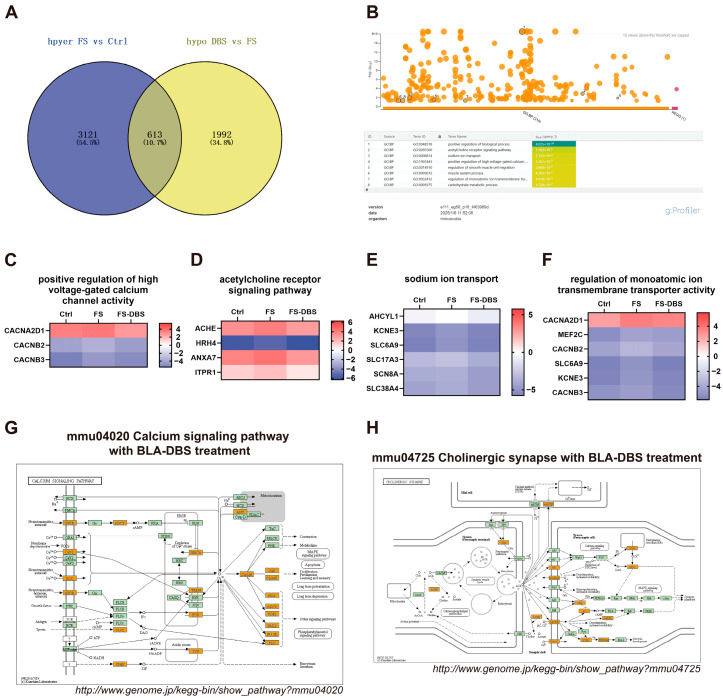
DBS attenuates the hypermethylation of m^6^A methylation caused by FS. (**A**) The Venn diagram illustrates the intersection of genes that are hypermethylated in the FS group compared to the Ctrl group and those that are hypomethylated in the DBS group compared to the FS group. (**B**) The biological processes GO enrichment diagram for the 613 intersecting genes obtained from the intersection in A was generated using the g:Profiler website. The gradient color scale indicates the level of GO enrichment, with a rise in color intensity signifying a greater enrichment. (**C**–**F**) Heatmap using the log2-scaled values of genes enriched in the acetylcholine receptor signaling pathway (**C**), positive regulation of high voltage-gated calcium channel activity (**D**), regulation of monoatomic ion transmembrane transporter activity (**E**), and sodium ion transport (**F**). (**G**,**H**) The screened differentially expressed genes were enriched in the calcium signaling pathway (**G**) and the cholinergic synapse pathway (**H**). Orange-marked nodes were associated with upregulated genes (FC > 1.5), whereas green nodes had no significance.

To further elucidate the regulatory effects of FS and BLA DBS on the m^6^A methylation of specific mRNAs, we analyzed mRNAs with hypermethylation in the FS vs. Ctrl comparison and hypomethylation in the FS-DBS vs. FS comparison. We found that the number of m^6^A-hypermethylated mRNAs in the FS vs. Ctrl comparison was 3734, while the number of hypomethylated mRNAs in the FS-DBS vs. FS comparison was 2605. By intersecting these two sets, we identified 613 overlapping mRNAs, representing 10.7% of the total mRNAs (Figure 4A). Using g:Profiler, we performed GO enrichment analysis on the 613 genes for biological processes and identified neuroscience-related pathways, including the acetylcholine receptor signaling pathway, sodium ion transport, the positive regulation of high voltage-gated calcium channel activity, and the regulation of monoatomic ion transmembrane transporter activity (Figure 4B). Using the log2-scaled values of mRNAs enriched in these pathways, we generated a heatmap and excluded genes with potential errors. We found that DBS effectively reversed the FS-induced m^6^A hypermethylation of mRNAs of several key genes in critical pathways. These genes included *Cacna2d1*, *Cacnb2*, and *Cacnb3* in the positive regulation of high voltage-gated calcium channel activity (Figure 4C); *Ache*, *Hrh4*, *Anxa7*, and *Itpr1* were involved in acetylcholine receptor signaling (Figure 4D); *Ahcyl1*, *Kcne3*, *Slc6a9*, *Slc17a3*, *Scn8a*, and *Slc38a4* were involved in sodium ion transport (Figure 4E); and *Cacna2d1*, *Mef2c*, *Cacnb2*, *Slc6a9*, *Kcne3*, and *Cacnb3* were involved in the regulation of high voltage-gated calcium channel activity (Figure 4F).

In the overlap between the FS vs. Ctrl and FS-DBS vs. FS comparisons, the integrated analysis of m^6^A methylation, GO enrichment, and gene expression results identified pathways in the KEGG database. We found that genes associated with the calcium signaling pathway (*Gpcr*, *Cav1*, *Roc*, *Plcζ*, *Sphk*, *Serca*, *Trdn*, *Ip3r*, *Tcp*, *Calm*, *Can Camk*, *Nos*, *Adcy*, *Pde1 Fak2*, *Ip3k*, *Pkc*) and cholinergic synapse pathway (*Ache*, *M1*, *Kir2*, *Nachr*, *Vgcc*, *Gi/O*, *Machr*, *Ac*, *Pi3k*, *Camk*, *Pkb/Akt*, *Pkc*) were upregulated, likely due to DBS-mediated reduction in methylation levels and subsequent increase in gene expression (Figure 4G,H).

### 3.5. BLA DBS Enhanced m^6^A Hypomethylation Caused by FS

**Figure 5:**
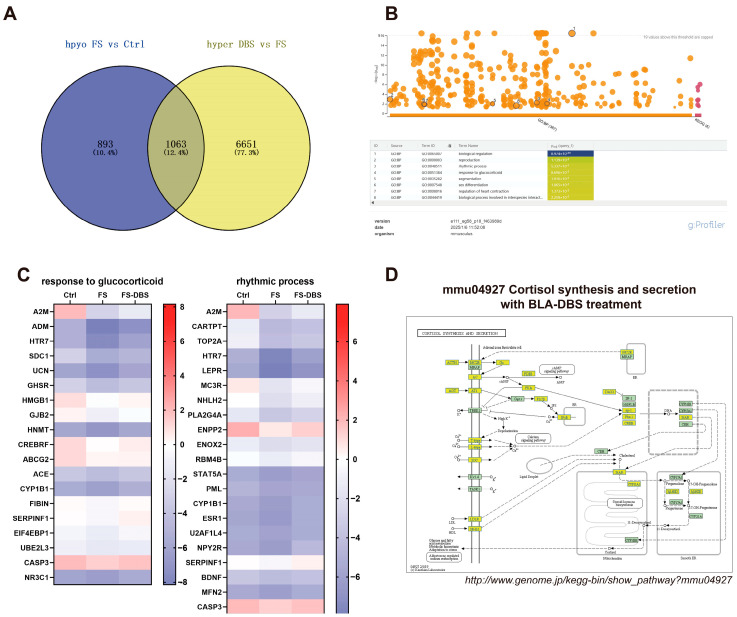
DBS enhances the hypomethylation of m^6^A methylation caused by FS. (**A**) The Venn diagram illustrates the intersection of genes that are hypomethylated in the FS group compared to the Ctrl group and those that are hypermethylated in the DBS group compared to the FS group. (**B**) The biological processes GO enrichment diagram for the 1063 intersecting genes obtained from the intersection in A was generated using the g:Profiler website. The gradient color scale indicates the level of GO enrichment, with a rise in color intensity signifying a greater enrichment. (**C**) Heatmap using the log2-scaled values of genes enriched in response to glucocorticoid (left) and the rhythmic process (right). (**D**) The screened differentially expressed genes were enriched in the cortisol synthesis and secretion pathway. Yellow-marked nodes were associated with downregulated genes (FC < −1.5), whereas green nodes had no significance.

Additionally, we analyzed the m^6^A-hypomethylated mRNAs in the FS vs. Ctrl comparison and the m^6^A-hypermethylated mRNAs in the DBS vs. FS comparison. After removing redundancy, 1956 m^6^A-hypomethylated mRNAs were identified in the FS group compared to the Ctrl group, and 7714 hypermethylated mRNAs were detected in the FS-DBS group relative to the FS group. By intersecting these two datasets, we identified 1063 mRNAs, representing 12.4% of the total mRNA count (Figure 5A). GO enrichment analysis of these 1063 mRNAs using g:Profiler highlighted neuroscience-related pathways, including the rhythmic process and response to glucocorticoids. Heatmaps were constructed based on the log2-scaled values of genes enriched in these pathways, with the careful exclusion of mRNAs containing inaccurate data (Figure 5B). We found that DBS corrected the hypomethylation of m^6^A in mRNAs of key genes caused by FS in the rhythmic process, including *A2m*, *Cartpt*, *Top2a*, *Htr7*, *Lepr*, *Mc3r*, *Nhlh2*, *Pla2g4a*, *Enpp2*, *Enox2*, *Rbm4b*, *Stat5a*, *Pml*, *Cyp1b1*, *Esr1*, *U2af1l4*, *Npy2r*, *Serpinf1*, *Bdnf*, *Mfn2*, and *Casp3* (Figure 5C), as well as those involved in the response to glucocorticoids, including *A2m*, *Adm*, *Htr7*, *Sdc1*, *Ucn*, *Ghsr*, *Hmgb1*, *Gjb2*, *Hnmt*, *Crebrf*, *Abcg2*, *Ace*, *Cyp1b1*, *Fibin*, *Serpinf1*, *Eif4ebp1*, *Ube2l3*, *Casp3*, and *Nr3c1* (Figure 5C).

We constructed relevant pathways using KEGG, focusing on the pathogenesis of PTSD, particularly the HPA axis dysregulation. The downregulation of genes associated with cortisol synthesis and secretion was observed, including *Acth*, *Mc2r*, *Agt*, *At1*, *T-Type*, *L-Type*, *Soc*, *Ldlr*, *Sr-B1*, *Gs*, *Pde8*, *Pka*, *Plcβ*, *Ip_3_r*, *Dax1*, *Sp-1*, *Pbx-1*, *Creb*, *Star*, *Cyp11a1*, and *3β-hsd* (Figure 5D). DBS-induced increases in the methylation levels may lead to the downregulation of this gene, indicating that DBS could achieve its therapeutic effects by modulating the response to glucocorticoids.

### 3.6. Detection of PTSD-Related Genes Using MeRIP Real-Time Quantitative PCR

The mechanisms of DBS are diverse, involving regulatory effects on neuronal excitability and synaptic plasticity [41]. Therefore, by using calcium signaling regulation and synaptic plasticity as key search terms, we systematically analyzed the data from the FS-DBS vs. FS comparison. Specifically, in calcium signaling-related genes, DBS intervention induced significant alterations in the methylation status of multiple critical genes, including *Adra1b* encoding alpha-1B adrenergic receptor [42], *Cacna2d2* encoding voltage-gated calcium channel auxiliary subunit alpha2delta-2 [43], and *Nts* and *Ntsr2* encoding neurotensin and its receptor [44]. For synaptic plasticity-related genes, we observed that DBS significantly altered the methylation levels of *Synj2* encoding synaptojanin 2 [45], *Npy6r* encoding neuropeptide Y receptor type 6 [46], and *Cnr1* and *Cnrip1* encoding cannabinoid receptor 1 and its interacting protein [47].

**Figure 6:**
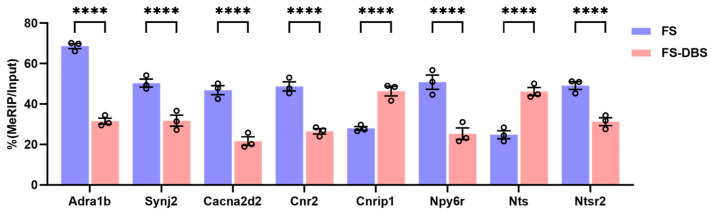
The detection of PTSD-related genes using MeRIP real-time quantitative PCR. Compared with the FS group, the DBS group exhibited the hypermethylation of *Cnrip1* and *Nts* and the hypomethylation of *Adra1b*, *Synj2*, *Cacna2d2*, *Cnr2*, *Npy6r*, and *Ntsr2* (two-way ANOVA, **** *p* < 0.0001).

To further confirm whether BLA DBS specifically regulates the methylation modification of the above genes, we verified these genes using Methylated RNA Immunoprecipitation (MeRIP)-qPCR. Compared with the FS group, the DBS group exhibited hypermethylation of the *Cast*, *Lepr*, *Nts*, and *Cnrip1* genes and hypomethylation of the *Adra1b*, *Trpm1*, *Synj2*, *Cacna2d2*, *Cnr2*, *Npy6r*, and *Ntsr2* genes. Thus, DBS may eliminate fear memories and treat PTSD by altering the methylation of neuroactivity and synapse plasticity in the vHPC through genes involved in acetylcholine signaling, calcium channel activity, and sodium ion transport (Figure 6). These results indicate that DBS not only reversed the m^6^A modification level of certain genes in response to FS, but also specifically influenced the m^6^A modification of several key genes associated with circuit plasticity in the vHPC.

## 4. Discussion

This study explored the epitranscriptomic mechanisms underlying the therapeutic effects of BLA DBS in a mouse model of PTSD. It revealed significant alterations in the m^6^A methylation levels in the vHPC. Our findings demonstrate that DBS alleviates PTSD-like behaviors induced by FS, which may be achieved through the dynamic modulation of mRNA methylation to restore the dysregulated molecular pathways in the vHPC. These results advance our understanding of how epigenetic regulation in the vHPC is involved in the PTSD pathological process and how BLA DBS ameliorates neuropsychiatric disorders.

We performed a correlation analysis between gene expression and epigenetic modifications and found that the “m^6^A methylation level”, calculated as the percentage of modified RNA per transcript, did not show a clear trend in correlation with gene expression (Figure S2A,B). In contrast, the “m^6^A quantity”, which is comparable across different samples, exhibited consistent upregulation or downregulation patterns in relation to gene expression (Figure S2C,D). Since the “m^6^A methylation levels” represent the overall percentage of RNA modification and the “m^6^A quantity “ refers to the methylation amount per transcript, we employed the “m^6^A quantity” for subsequent analysis to enable direct transcript-to-transcript comparisons.

The analysis showed that DBS plays a dual regulatory role in reducing the FS-induced m^6^A hypermethylation of mRNAs and enhancing the FS-induced m^6^A hypomethylation of mRNAs in the vHPC. It may specifically regulate the m^6^A modifications of genes related to acetylcholine signaling (e.g., *Ache*, *Hrh4*), calcium channel activity (e.g., *Cacna2d1*, *Cacnb2*), and sodium ion transport (e.g., *Scn8a*) to modify synaptic plasticity and neuronal excitability for fear extinction and emotional regulation. As reported in the literature, the activity changes in vHPC neurons are closely associated with the formation and retrieval of fear memory [48], while the enhanced synaptic plasticity of inhibitory neurons promotes fear memory extinction [49,50]. Furthermore, DBS may regulate the dysregulation of glucocorticoid-responsive genes caused by stress, such as *Nr3c1*, indicating its potential to regulate the HPA axis activity, a crucial aspect of PTSD. Studies have revealed that PTSD patients often exhibit the dysregulation of the HPA axis, characterized by reduced cortisol levels and increased glucocorticoid receptor (GR) sensitivity [51,52]. Nonetheless, there is evidence that a subset of PTSD patients may display blunted responses in corticotropin-releasing hormone (CRH) activity and GR sensitivity [53]. Our findings suggest that BLA DBS may reduce GR sensitivity, which could represent one of the mechanisms underlying its efficacy in PTSD treatment.

Due to the complexity of PTSD and the limitations of animal models of PTSD, future studies should investigate DBS effects and m^6^A modifications in additional models. The conservation of key stress-related genes (e.g., *Bdnf*) between murine models and humans enhances the translational potential of our findings. These results provide novel insights into the role of m^6^A methylation in clinical research, creating new avenues for therapeutic intervention.

Our validation experiments revealed that DBS altered the methylation levels of specific genes in the FS group mice, whereas FS alone did not induce such changes. This suggests that the therapeutic effects of DBS are not merely mediated through reversing FS-induced modifications, but may also involve the direct regulation of neural function. However, the underlying mechanisms appear to be complex, as evidenced by the DBS-induced hypermethylation of *Cnrip1* and *Nts* genes, coupled with the hypomethylation of *Cnr2* and *Ntsr2* genes. These coordinated epigenetic modifications indicate that DBS may influence synaptic plasticity through a sophisticated regulatory mechanism.

However, this study has several limitations that warrant consideration. First, the pooled mRNA samples from multiple mice may obscure individual variability. Second, the exclusive use of male mice limits generalizability to females, given the known sex differences in PTSD susceptibility and epigenetic regulation. Third, the short-term DBS protocol does not address the potential long-term adaptive changes or relapse risks. Translational efforts could also integrate neuroimaging [54] or biomarker analyses [55] to bridge preclinical findings with clinical DBS applications.

## 5. Conclusions

In summary, this study proves that BLA DBS alleviates PTSD-like behaviors via m^6^A modifications in the vHPC. By correlating m^6^A dynamics with PTSD models and BLA DBS treatments, we propose the involvement of several genes associated with mRNA methylation and several pathways in PTSD development and the effects of DBS. Our findings reveal that DBS exerts its therapeutic effects not only by reversing the aberrant mRNA methylation of key genes in PTSD-related pathways induced by FS, but also by modulating the methylation levels of critical genes associated with synaptic plasticity. This suggests that alterations in m^6^A modification may provide novel research directions and therapeutic strategies for improving clinical PTSD symptoms. BLA DBS treatment induced m^6^A modifications in the vHPC, suggesting that BLA DBS could exhibit a therapeutic effect through the synergistic regulation of downstream targets of BLA. These findings provide valuable insights into the development of diverse PTSD intervention targets, as well as new molecular intervention targets besides DBS.

## Data Availability

The m^6^A transcriptome sequencing data are available in the following website: https://doi.org/10.6084/m9.figshare.28631330.

## Supplementary Materials

The following supporting information can be downloaded at: https://www.mdpi.com/article/10.3390/brainsci15050473/s1. Figure S1: Alterations in the vHPC gene expression levels. (A–C) Heatmap showing genes expressionlevel changed by at least 1.5-fold in FS group compared with Ctrl group (A), DBS group compared with Ctrl group (B) and DBS group compared with FS group (C). (D–F) Pie chart illustrating the upregulated and downregulated genes across (A–C); Figure S2: Correlation analysis of gene methylation and gene expression in the vHPC. (A,B) Methylation(MethylationLevel) associated with gene expression in DBS group compared with FS gropup (A) and FS group compared with Ctrl gropup (B). Green dots represent hypermethylation genes associated with downregulated gene expression, red dots represent hypermethylation genes associated with upregulated gene expression, blue dots represent hypomethylation genes associated with downregulated gene expression, and purple dots represent hypomethylation genes associated with upregulated gene expression (m^6^A-hyper/gene-up: FC > 1.5; m^6^A-hypo/gene-down: FC < −1.5). (C,D) Methylation(Quantity) associated with gene expression in DBS group compared with FS gropup (C) and FS group compared with Ctrl gropup (D); Table S1: Specific data for Figure 3.
