# Supplementary material for: Epitranscriptomic Analysis of the Ventral Hippocampus in a Mouse Model of Post-Traumatic Stress Disorder Following Deep Brain Stimulation Treatment of the Basolateral Amygdala
Source: Brain Sci. 2025 Apr 29;15(5):473. doi: 10.3390/brainsci15050473 (PMC12109718; doi:10.3390/brainsci15050473)
Supplement: Supplementary file 1 [file brainsci-15-00473-s001.zip › supplementary materials-4.28.pdf]

Epitranscriptomic Analysis of the Ventral Hippocampus in a Mouse Model of Post-Traumatic Stress Disorder Following Deep Brain Stimulation Treatment of the Basolateral Amygdala Supplementary material

1 Supplementary methods

1.1 Fear conditioning test

Upon being reintroduced to a chamber where they had previously experienced foot shocks, mice exhibit intermittent freezing behavior as a manifestation of fear. The duration of freezing behavior during a 5-minute test was recorded and analyzed using PACKWIN software (Panlab, Spain), which quantified the percentage of time the mice remained immobile.

1.2 Magnetic Resonance Imaging (MRI)

Following anesthesia induction with 2.5-3.0% isoflurane, animals were positioned in a customized head restraint system incorporating an integrated radiofrequency coil and body stabilization tube. Anesthesia maintenance employed 1.5-2.0% isoflurane delivered via nasal interface within a 30% oxygen/70% nitrogen carrier gas mixture, with the entire assembly subsequently positioned within the magnetic bore. Continuous physiological monitoring included respiratory frequency and core temperature tracking using a PC-SAM Small Animal Monitor (Small Animal Instruments Inc.), with automated thermal regulation maintaining body temperature at 37.0 ± 0.5°C through a feedback-controlled heating platform (SC100-S14P, Thermo Scientific).

Structural and functional neuroimaging was performed using a 7.0 T preclinical MRI system (Bruker BioSpin, Germany) equipped with a 50 mm mouse brain quadrature surface coil. Anatomical reference images were acquired through T2-weighted TurboRARE sequencing with optimized parameters: TR/TE = 4500/35 ms, 0.35 mm slice thickness (0.05 mm interslice gap), 20×20 mm^2 FOV, 256×256 matrix, 152.587 kHz bandwidth, and 40 contiguous axial slices. Resting-state functional MRI data acquisition and system control were implemented through Paravision 6.0.1 software (Bruker BioSpin), ensuring standardized protocol execution across experimental sessions.

The preprocessing of imaging data was performed using SPM12 software (http://www.fil.ion.ucl.ac.uk/spm/software/spm12/) to correct for head motion and spatial alignment through co-registration with anatomical reference images. Spatial smoothing was subsequently applied using a Gaussian kernel to enhance the signal-to-noise ratio across all slices. For functional connectivity analysis, regions of interest (ROIs) were defined by selecting 4-5 voxels within the vHPC in each subject. This analysis was conducted using REST software (http://restfmri.net/forum/index.php) in conjunction with custom Matlab2014a scripts (www.mathworks.com). Between-group comparisons of functional connectivity patterns (twelve mice per group) were

conducted by computing the differential connectivity values, with statistical significance assessed through two-sample *t*-tests.

1.3 M6A-mRNA data analysis

The scanned microarray images were imported into Agilent Feature Extraction software for raw data extraction.

The "m6A methylation level" for a transcript was calculated as the percentage of modified RNA (%Modified) in all RNAs based on the IP (Cy5-labelled) and Sup (Cy3-labelled) normalized intensities:

$$\%\text{Modified} = \frac{\text{modified RNA}}{\text{Total RNA}} = \frac{\text{IP}}{\text{IP} + \text{Sup}}$$
$$= \frac{\boldsymbol{IP}_{Cy5\ normalized\ intensity}}{\boldsymbol{IP}_{Cy5\ normalized\ intensity} + \boldsymbol{Sup}_{Cy3\ normalized\ intensity}}$$

Raw intensities of IP (immunoprecipitated, Cy5-labelled) and Sup (supernatant, Cy3-labelled) were normalized with average of log2-scaled Spike-in RNA intensities.

$$\log 2(\boldsymbol{IP}_{Cy5\ normalized\ intensity})$$
$$= \log 2(\boldsymbol{IP}_{Cy5\ raw}) - \text{Average}[\log 2(\boldsymbol{IP}_{spike-in\_Cy5\ raw})]$$

$$\log 2(\boldsymbol{Sup}_{Cy3\ normalized\ intensity})$$
$$= \log 2(\boldsymbol{Sup}_{Cy3\ raw}) - \text{Average}[\log 2(\boldsymbol{Sup}_{spike-in\_Cy3\ raw})]$$

The "m6A quantity" was calculated for the m6A methylation amount of each transcript based on the IP (Cy5-labelled) normalized intensities.

$$\text{SampleA m6A quantity} = \text{SampleA}\ \boldsymbol{IP}_{Cy5\ normalized\ intensity}$$

Raw intensities of IP (Cy5-labelled) were normalized by average of log2-scaled Spike-in RNA intensities.

$$\log 2(\boldsymbol{IP}_{Cy5\ normalized\ intensity})$$
$$= \log 2(\boldsymbol{IP}_{Cy5\ raw}) - \text{Average}[\log 2(\boldsymbol{IP}_{spike-in\_Cy5\ raw})]$$

The "Expression level" for transcript was calculated as the quantity of all RNAs (modified and not modified RNA) based on the IP (Cy5-labelled) and Sup (Cy3-labelled) normalized intensities:

$$\text{Expression level of Total RNA} = \text{IP} + \text{Sup}$$
$$= \boldsymbol{IP}_{Cy5\ normalized\ intensity} + \boldsymbol{Sup}_{Cy3\ normalized\ intensity}$$

Raw intensities of IP (immunoprecipitated, Cy5-labelled) and Sup (supernatant, Cy3-labelled) were normalized with average of log2-scaled Spike-in RNA intensities.

$$\log 2(\boldsymbol{IP}_{Cy5\ normalized\ intensity}) = \log 2(\boldsymbol{IP}_{Cy5\ raw}) - \text{Average}[\log 2(\boldsymbol{IP}_{spike-in\_Cy5\ raw})]$$

$$\log 2(\boldsymbol{Sup}_{Cy3\ normalized\ intensity}) = \log 2(\boldsymbol{Sup}_{Cy3\ raw}) - \text{Average}[\log 2(\boldsymbol{Sup}_{spike-in\_Cy3\ raw})]$$

To compare two groups for differential m6A modification (i.e. disease vs. control), the fold change (FC) and statistical significance of the difference (p-value) were calculated for each transcript. The default thresholds are |FC| 1.5 without having replicated samples or |FC| > 1.5 and p-values ≤ 0.05 with having replicated samples. One can adjust the stringency thresholds and rank the differentially m6A-methylated RNAs further by using Microsoft Excel's Data/Sort & Filter functionalities.

To compare two groups for differential expression (i.e. disease vs. control), the fold change (FC) and statistical significance of the difference (p-value) were calculated for each transcript. The default thresholds are |FC| > 1.5 without having replicated samples or |FC| > 1.5 and p-values ≤ 0.05 with having replicated samples. One can adjust the stringency thresholds and rank the differentially expressed RNAs further by using Microsoft Excel's Data/Sort & Filter functionalities.

1.4 MeRIP real-time quantitative PCR

MeRIP-qPCR is designed to validate m^6A RNA methylation levels at specific loci identified via high-throughput sequencing (MeRIP-seq). Unlike conventional qPCR, which quantifies total RNA expression, MeRIP-qPCR focuses on the proportion of RNA molecules with a specific modification at a defined region. Results are expressed as % (MeIP/Input), reflecting the ratio of methylated RNA fragments (MeIP) to total RNA fragments (Input) for a target locus.

Total RNA (vHPC tissues pooled from 3 mice per group) is chemically or enzymatically fragmented into ~100-nt segments to ensure antibody accessibility. Fragmented RNA is split into two aliquots: IP Sample: Immunoprecipitated using anti-m6A. Input Sample: Unenriched control for normalization. Both IP and Input RNA are reverse-transcribed into cDNA. Target regions are amplified using locus-specific primers. Ct values are recorded for both samples.

Calculate the % Input for each MeRIP fraction:

$$\%\text{Input} = \frac{2^{-\text{Ct MeRIP}}}{2^{-\text{Ct MeRIP}} + 2^{-\text{Ct Supernatant}}} \times \text{Fd} \times 100\%$$

Here, Fd is Input dilution factor. For example, if one of supernatant RNA and one of MeRIP RNA were used for qPCR assays, Fd =1/1.

2 Supplementary data

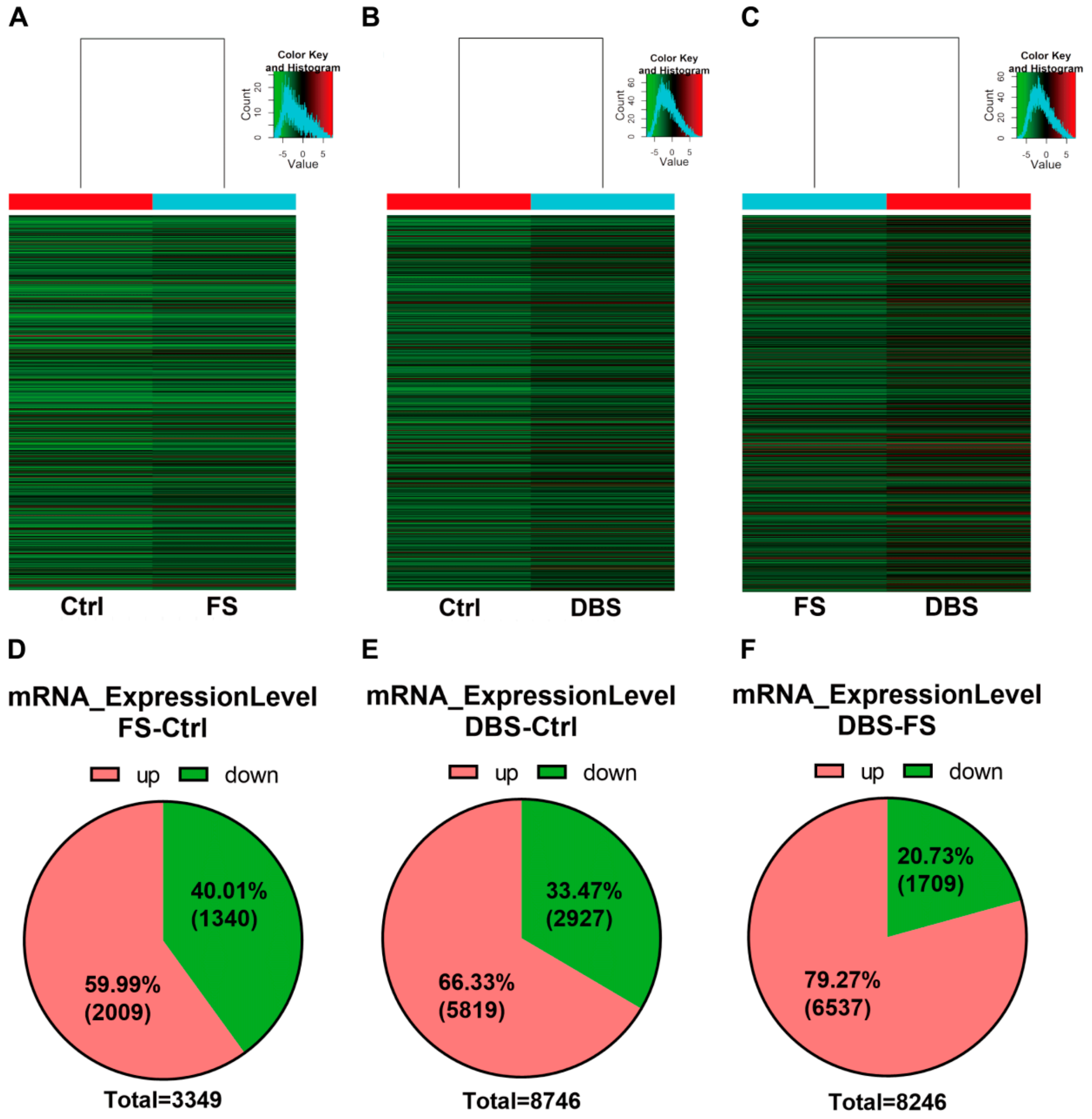

Figure S1: Alteration of vHPC gene expression levels. (A-C) Heatmap showing genes expressionlevel changed by at least 1.5-fold in FS group compared with Ctrl group (A), DBS group compared with Ctrl group (B) and DBS group compared with FS group (C). (D-F) Pie chart illustrating the upregulated and downregulated genes across A-C.

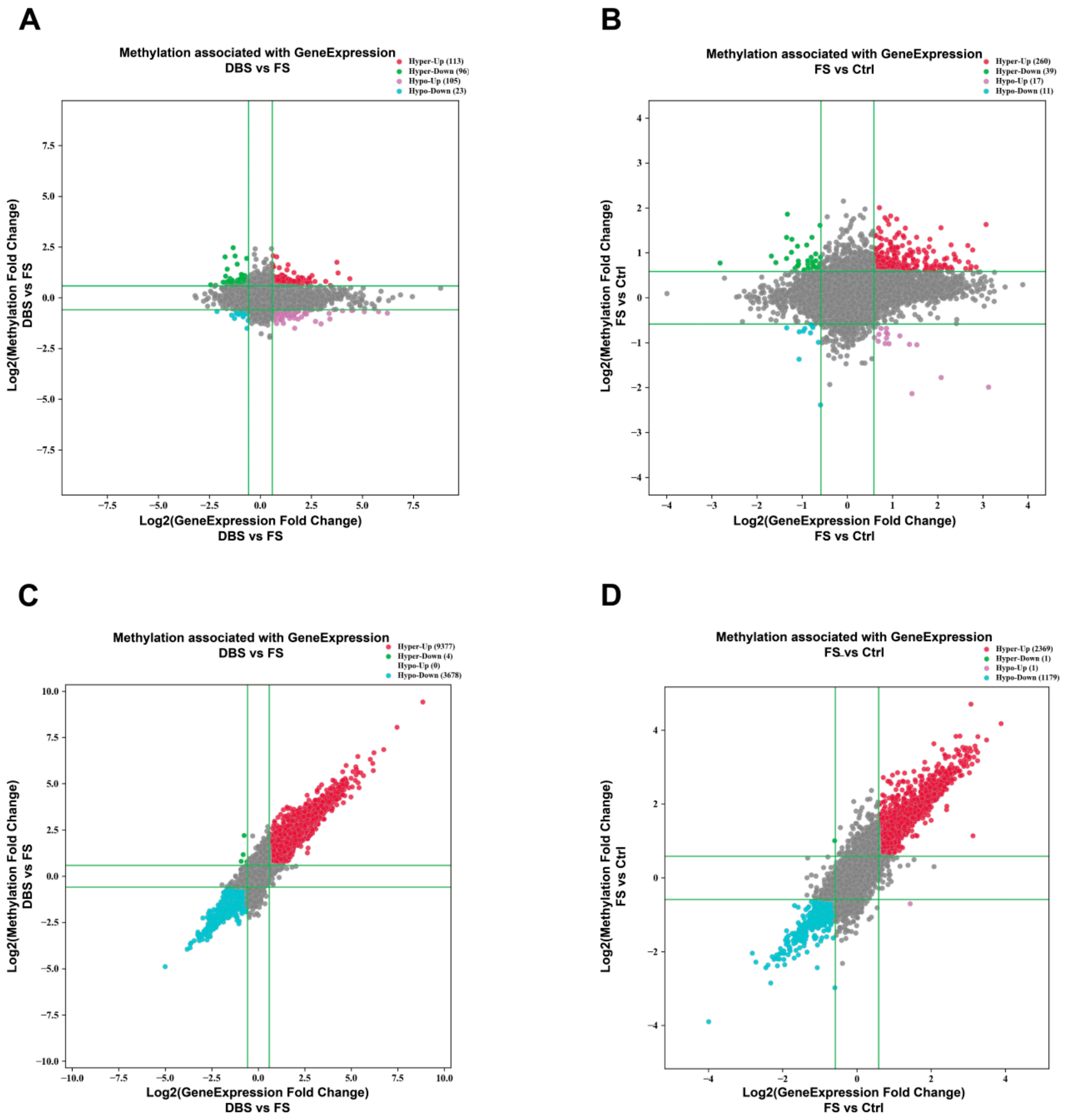

Figure S2: Correlation analysis of gene methylation and gene expression in the vHPC. (A-B) Methylation(MethylationLevel) associated with gene expression in DBS group compared with FS gropup (A) and FS group compared with Ctrl gropup (B). Green dots represent hypermethylation genes associated with downregulated gene expression, red dots represent hypermethylation genes associated with upregulated gene expression, blue dots represent hypomethylation genes associated with downregulated gene expression, and purple dots represent hypomethylation genes associated with upregulated gene expression (m^6A-hyper/gene-up: FC > 1.5; m^6A-hypo/gene-down: FC < -1.5). (C-D) Methylation(Quantity) associated with gene expression in DBS group compared with FS gropup (C) and FS group compared with Ctrl gropup (D).
